# Human Metapneumovirus Infections in Hospitalized Children^1^

**DOI:** 10.3201/eid0906.030017

**Published:** 2003-06

**Authors:** Guy Boivin, Gaston De Serres, Stéphanie Côté, Rodica Gilca, Yacine Abed, Louis Rochette, Michel G. Bergeron, Pierre Déry

**Affiliations:** * ^Centre Hospitalier Universitaire de Québec, Québec City, Québec, Canada^; † Laval University, Québec City, Québec, Canada; ‡ Québec Institute of Public Health, Québec City, Québec, Canada

**Keywords:** Human metapneumovirus, human respiratory syncytial virus, respiratory tract infections, children, real-time PCR, research

## Abstract

We evaluated the percentage of hospitalizations for acute respiratory tract infections in children <3 years of age attributable to human metapneumovirus (HMPV) and other respiratory viruses in a prospective study during winter and spring 2002. We used real-time polymerase chain assays and other conventional diagnostic methods to detect HMPV, human respiratory syncytial virus (HRSV), and influenza viruses in nasopharyngeal aspirates of children. HMPV was detected in 12 (6%) of the 208 children hospitalized for acute respiratory tract infections, HRSV in 118 (57%), and influenza A in 49 (24%). Bronchiolitis was diagnosed in 8 (68%) and pneumonitis in 2 (17%) of HMPV-infected children; of those with HRSV infection, pneumonitis was diagnosed in 99 (84%) and bronchiolitis in 30 (25%). None of the HMPV-infected children was admitted to an intensive-care unit, whereas 15% of those with HRSV or influenza A infections were admitted. HMPV is an important cause of illness in young children with a similar, although less severe, clinical presentation to that of HRSV.

The human metapneumovirus (HMPV) is the first member of the new *Metapneumovirus* genus (*Paramyxoviridae* family) that infects humans (*1*,*2*). The human respiratory syncytial virus (HRSV) belongs to a separate genus within the same family (*3*). HMPV has been recently identified in nasopharyngeal aspirates of children and adults with acute respiratory tract infections (ARTI) in various parts of the world (*1*,*4*–*7*). The clinical syndrome of the infected children ranges from mild respiratory problems to bronchiolitis and pneumonitis (*1*,*7*–*9*).

When reverse-transcription polymerase chain reaction (RT-PCR) has been used, the proportion of HMPV detected in nasopharyngeal aspirate samples from children with unexplained ARTI has varied from 1.5% to 10% (*1*,*4*,*7*). However, most retrospective studies had limitations: for example, they were small, excluded patients who tested positive for other viruses, only superficially described the clinical features of the disease, and lacked data on illness severity and death. Moreover, in the absence of a control group, these studies could not differentiate whether HMPV was a colonizing or a pathogenic virus. More recently, Stockton et al. identified HMPV RNA in 2.2% of 405 specimens from patients with influenzalike illnesses who consulted general practitioners in England, although few swabs were collected from children <5 years of age (*6*).

The objectives of this study were to estimate the relative contribution of HMPV in children’s hospitalization for ARTI and to define its clinical features and seasonal pattern relative to other common respiratory viruses over a single winter season.

## Materials and Methods

### Study Design

Participants were children <3 years of age who were hospitalized from December 15, 2001, to April 20, 2002, at Laval University Hospital Center in Québec City, Québec, Canada. Case-patients were children admitted for an ARTI (mostly bronchiolitis, pneumonitis, and laryngotracheobronchitis) who had a nasopharyngeal aspirate collected as part of the investigation of their illness (in this hospital, collecting such samples is standard practice to assess the presence of HRSV in children). The research nurse at the microbiology laboratory that received the nasopharyngeal aspirate specimens identified eligible case-patients. Case-patients hospitalized twice were counted as two cases. A specific questionnaire for the study was completed at admission by a single research nurse with the parents. At the end of the hospitalization, the children’s charts were reviewed to collect clinical and laboratory data by using a standardized protocol. Eligible controls were children hospitalized for any elective surgery who had no respiratory symptoms or fever. At admission, the nurse obtained a signed consent from parents and collected a nasopharyngeal aspirate (1–2 mL). The study was approved by the Centre Hospitalier Universitaire de Québec research ethics board.

### Laboratory Testing

For this study, all specimens from case-patients and controls were tested by RT-PCR for HMPV, influenza A and B, and HRSV. Antigen detection for HRSV was performed for all case-patients immediately at admission. Viral cultures and other antigen detection assays were performed on request of the treating physician. The rest of the specimen was then frozen at –80°C until subsequent RT-PCR studies.

### RNA Extraction and RT-PCR Studies

Viral RNA was extracted from 200 μL of nasopharyngeal aspirate specimens by using the QIAamp viral RNA Mini Kit (QIAGEN, Inc., Mississauga, ON, Canada). Complementary cDNA was synthesized by using 10 μL of eluted RNA and the Omniscript Reverse Transcriptase (QIAGEN). Random hexamer primers (Amersham Pharmacia Biotech, Baie d’Urfé**,** Québec, Canada) were used in the RT step of all PCR assays, except for HMPV, in which a specific primer (5′-TGGGACAAGTGAAAATGTC-3′) served to synthesize HMPV cDNA. PCR assays were designed to amplify conserved regions of influenza A (*10*), influenza B (*11*), and HRSV (*12*) genes. New PCR primers were designed for amplification of the HMPV N (nucleoprotein) gene. The sequences of the forward and reverse primers were respectively 5′-GAGTCTCAGTACACAATTAA-3′ and 5′-GCATTTCCGAGAACAACAC-3′. Complementary DNA was amplified for all respiratory viruses by using a standardized RT-PCR protocol with the LC Faststart DNA Master SYBR Green 1 Kit (Roche Diagnostics, Laval, Québec, Canada) in a LightCycler instrument (Roche Diagnostics). The melting curve analysis program of the LightCycler was used to identify specific PCR products. Each PCR assay could detect at least 50 copies of viral target. For phylogenetic studies, nucleotide sequences were determined from amplified HMPV F (fusion) gene products, then analyzed by using the neighbor-joining algorithm and Kimura-2 parameters (*9*).

### Standard Viral Cultures and Antigenic Assays

Specimens were injected onto 96-well plates containing 10 cell lines (MDCK, LLC-MK2, Hep-2, human foreskin fibroblast, Vero, mink lung, A-549, rhabdomyosarcoma, 293, and HT-29) and then incubated for 21 days. A positive cytopathic effect was confirmed by immunofluorescence testing with monoclonal antibodies or by RT-PCR (HMPV) (*9*). Detection of HRSV and influenza antigens was performed directly on nasopharyngeal aspirate samples by using commercially available immunoenzymatic assays (RSV TestPack, Abbott Laboratories, Abbott Park, IL; Directigen Flu A + B, Becton Dickinson Microbiology Systems, Sparks, MD). Viral antigens for adenoviruses and parainfluenza viruses 1–3 were sought in specimens by an immunofluorescence method with specific monoclonal antibodies (*9*).

### HMPV in the General Population

To further assess the seasonal distribution, affected age groups, and frequency of HMPV, we compared data from this study with data from the general population using positive viral cultures reported by our regional virology diagnostic laboratory, the only one performing viral cultures for the Québec City area (population 600,000). Isolation of HMPV was achieved by observing typical cytopathic effect on LLC-MK2 cells, followed by PCR confirmation on infected cell culture supernatants (*9*).

### Statistical Analyses

The Wilcoxon nonparametric test was used to compare the age distribution of case-patients and controls and period of hospitalization. The proportion of cases and controls with HMPV infections and the clinical features of children infected with HMPV versus those infected with other respiratory viruses were compared by the χ^2^ test or the Fisher exact test. Analyses were performed by using SAS software version 8.02 (SAS Institute, Cary, NC).

## Results

### Study Population and Viral Etiologic Agents

**Figure 1:**
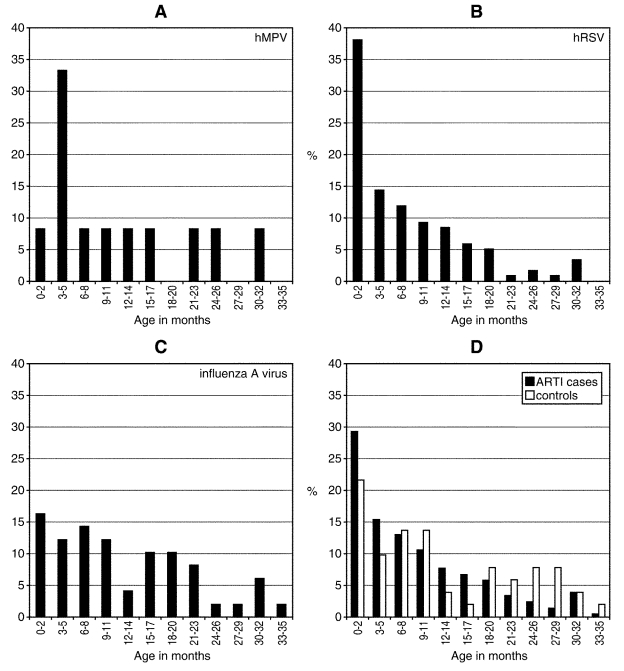
Age at admission of children hospitalized for acute respiratory tract infections caused by human metapneumovirus (HMPV) (A), human respiratory syncytial virus (HRSV) (B), and influenza A (C) as well as for the whole study population (D).

**Figure 2:**
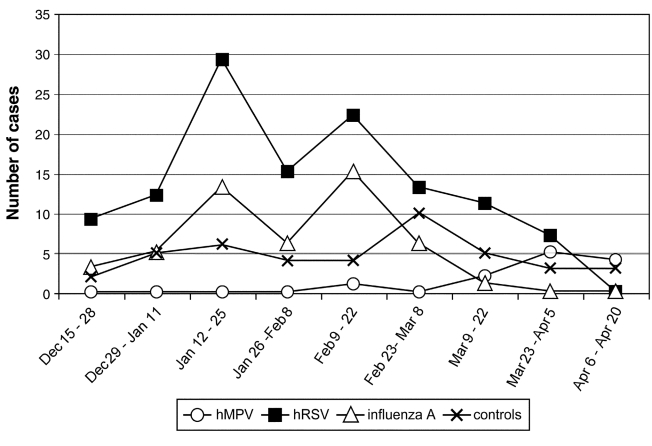
Biweekly distribution of virologically confirmed cases with acute respiratory tract infections and their controls.

The study population included 208 hospitalized case-patients with ARTI (including 8 children who were admitted twice) and 51 children who served as controls. The age distribution of case-patients and controls is presented in Figure 1. Infants ≤3 months of age were most likely to be hospitalized, and the rate of hospitalizations steadily decreased in children >3 months. The mean age was slightly younger for case-patients than for controls (mean 9 months vs. 12 months, Wilcoxon test p=0.06). Among cases with ARTI, 56% were male as were 57% of controls (p=0.88). The date of hospitalization was similar for case-patients and controls (p=0.84) (Figure 2). Most children (90%) had no underlying medical conditions at admission.

A nasopharyngeal aspirate sample was taken for all 208 case-patients and 51 controls. For case-patients, the mean delay between the onset of symptoms and collection of nasopharyngeal aspirates was 6 days (median 4 days) (Table 1). This delay did not differ for the different viruses detected. Samples from all 208 case-patients were tested by PCR for HMPV, HRSV, and influenza A and B; 204 samples were tested for HRSV antigen; 172 were assayed for other viral antigens; and 145 were tested by viral culture for the whole panel of respiratory viruses (as ordered by the treating physician). At least one respiratory virus was detected by one of the above methods in 164 (78.8%) cases, whereas none was detected in 44 (21.1%). Combining these diagnostic techniques, 12 cases (5.8%) were positive for HMPV, 118 (56.7%) for HRSV, 49 (23.6%) for influenza A, and none for influenza B (Table 1). In contrast, a virus was not detected by PCR in any of the control samples (p=0.067 for HMPV, one-sided Fisher exact test). PCR testing was not done for adenoviruses and parainfluenza viruses, but these viruses were respectively found in 6/145 (4.1%) and 2/145 (1.3%) of tested case-patients by the use of viral cultures or antigenic assays. Single virus infections occurred in 141 (86.0%) of the 164 positive case-patients, and mixed infection was found in 23 (14.0%). Two of the 12 HMPV infections were mixed (HMPV-influenza and HMPV-HRSV). The other combinations were HRSV–influenza A (18 cases), HRSV-adenovirus (*2*), and influenza A–adenovirus (*1*).

**Table 1:** Type of laboratory confirmation by type of infection

Laboratory test	HMPV^a^		HRSV		Influenza A		Adenovirus		PIV 2		
	No. tests	Positive (%)	No. tests	Positive (%)	No. tests	Positive (%)	No. tests	Positive (%)	Tests done	Positive (%)	
PCR	208	12 (5.8)	208	106 (51.0)	208	45 (21.6)	NA	NA	NA	NA	
Culture	145	2 (1.4	145	37 (25.5)	145	10 (6.9)	145	6 (4.1)	145	1 (0.7%)	
Antigen detection	NA	NA	204	94 (46.1)	172	19 (11.0)	81	1 (1.2)	76	1 (1.3%)	
Total (+) in at least one test	12 (5.8%)		118 (56.7)		49 (23.6)		6 (4.1)		2 (1.3%)		
Delay between onset of symptoms and NPA, days; mean/median	6.3/5.0		5.2/4.0		8.7/5.0		6.0/6.5		3.0/3.0		

^a^HMPV, human metapneumovirus; HRSV, human respiratory syncytial virus; PIV, parainfluenza virus; NPA, nasopharyngeal aspirate; PCR, polymerase chain reaction; NA, not applicable.

Among the 208 case-patients tested by PCR for HMPV, HRSV, and influenza A and B viruses, the positivity rates were 5.8%, 51.0%, 21.6%, and 0%, respectively (Table 1). In addition, 16 other case-patients had one of these four respiratory viruses identified only by culture (one influenza A and one HRSV), only by an antigen detection test (nine HRSV and three influenza A), or by both culture and antigen detection test (two HRSV). Among the eight children who were hospitalized twice, none had the same viral infection at both admissions. The specific combinations observed were HMPV-HRSV (two), HMPV–no virus (one), HRSV–no virus (two), HRSV-influenza (two), no virus–no virus (one).

The biweekly distribution of cases with respiratory tract viruses is shown in Figure 2. HRSV and influenza A infections occurred predominantly from January to March, whereas HMPV infections occurred mostly in March and April. The proportion of children with virologically confirmed respiratory tract infections decreased after February.

### Clinical Features of Cases

Given the small number of HMPV cases, the results only suggest trends, as no statistical comparison reached significance. The peak age for hospitalized HMPV infection was 3–5 months, whereas it was 0–2 months for HRSV infection (Figure 1). Influenza A virus infection occurred evenly throughout the first year of life. The peak age for mixed infection was 6–11 months; the frequency of such infections decreased thereafter. Gender was distributed evenly within each virus group, but more males (70%) had mixed viral infections. Most (75% with HMPV, 93% with HRSV, 90% with influenza A virus infection) of the children in the etiologic agent groups had no underlying medical conditions. Three (25%) children with HMPV infection had a cardiac disorder, including one child with multiple medical problems.

Signs and symptoms recorded with the different respiratory viruses were similar (Table 2). The median duration of hospitalization was similar for HMPV, HRSV, and influenza A viruses being respectively 4.5, 5.0, and 4.0 days (p=0.85). Of note, four (33.3%) HMPV-infected children were hospitalized for >7 days, including one child with underlying conditions. None of the children with HMPV infection was admitted to the intensive-care unit (ICU) in contrast to 15% (p=0.22) with HRSV and 16% (p=0.34) with influenza A infections. None of the children in this study died. The duration of the hospitalization for children with no detectable virus was shorter than that for children with single or mixed infection (Wilcoxon test, p <0.001). Two thirds of the children were given antibiotics during their hospitalization, although almost none had specimens collected for bacterial cultures.

**Table 2:** Signs and symptoms by type of viral infection

Signs and symptoms	% HMPV, n=12	% HRSV, n=118	% Influenza A, n=49	% Single virus, n=141	% Multiple viruses, n=23	% No virus detected, n=44	% Total; n=208
Fever	67	57	78	60	74	57	61
Cough	100	99	96	98	100	90	97
Rhinorrhea	92	91	84	87	96	96	90
Retractions	92	95	82	89	96	89	89
Wheezing	83	65	57	59	83	71	64
Lacrymation	25	31	31	33	26	25	30
Diarrhea	8	17	27	17	22	23	19
Vomiting	25	8	10	7	17	2	7
Other	0	26	18	23	17	21	22

^a^HMPV, human metapneumovirus; HRSV, human respiratory syncytial virus. Given the small number of HMPV cases, the results only suggest trends, as no statistical comparison reached significance.

At hospital discharge, a final diagnosis of bronchiolitis was given to 67% of children with HMPV, 84% with HRSV, and 51% with influenza A (p <0.001) (Table 3). Otitis media occurred in about half of the children with HMPV, HRSV, and influenza A virus infections. Pneumonitis was less frequently diagnosed in children with HMPV compared to those with HRSV or influenza A (17%, 25%, and 37%; p=0.22). Definitive clinical diagnoses were similar with single and mixed infections.

**Table 3:** Definitive clinical diagnoses by type of viral infection

Complication	% HMPV,^a^ n=12	% HRSV, n=118	% Influenza A, n=49	% Single virus, n=141	% Multiple viruses, n=23	% No virus detected, n=44	% Total, n=208	
Bronchiolitis	67	84	51	70	83	57	68	
Pneumonia	17	25	37	28	30	27.	28	
Laryngotracheobronchitis	0	10	12	8	17	5	8	
Otitis	50	59	55	55	65	55	56	
Sinusitis	0	3	6	1	9	2	2	
Pharyngitis	0	1	0	1	0	5	2	
Flu syndrome	0	2	0	1	0	9	3	
Other	8	3	6	6	0	11	7	

^a^HMPV, human metapneumovirus; HRSV, human respiratory syncytial virus. Given the small number of HMPV cases, the results only suggest trends, as no statistical comparison reached significance.

### HMPV in the General Population

**Figure 3:**
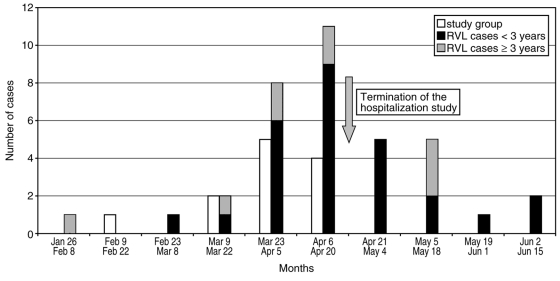
Biweekly distribution of virologically confirmed human metapneumovirus (HMPV) cases from the prospective pediatric study (study group) and from the general population as retrospectively identified in the Québec City Regional Virology Laboratory (RVL).

The regional virology laboratory received 1,505 respiratory specimens for viral culture from January 1 to June 30, 2002. In total, 36 (including 2 study participants) or 2.9% were positive for HMPV: 24 (67%) in children <2 years of age, 5 (14%) in those 2 to 4 years of age, 4 (11.1%) in adults 30–49 years of age, and 3 (8%) in those >70 years of age. No clinical information was available from these cases. Most isolates (81%) were recovered over a 2-month period (from March 23 to May 18 ). When the seasonal distribution of HMPV in hospitalized children (study population) and in the seasonal distribution in the general population were compared, we found that the study did not cover the entire HMPV season and that it had been stopped just after the peak time of HMPV transmission (April 6–20) (Figure 3).

### Phylogenetic Analyses of HMPV Strains

**Figure 4:**
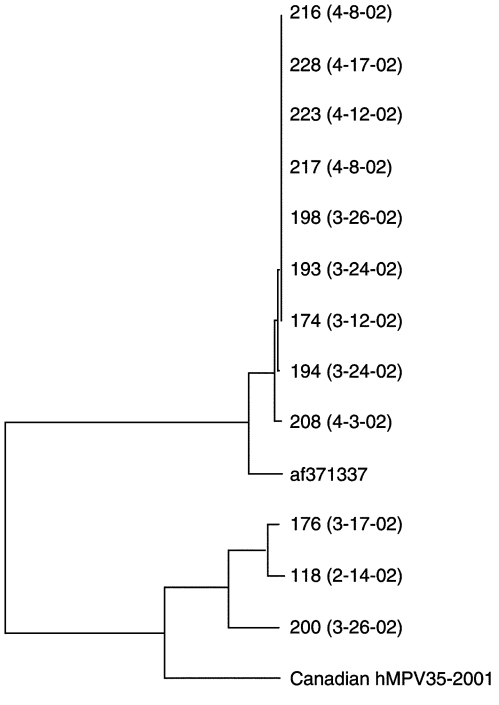
Phylogenetic tree showing sequence analysis of the F (fusion) gene of 12 human metapneumovirus (HMPV) strains detected in 2002 as part of this study and of the prototype strain from the Netherlands (GenBank accession no. af371337) as well as from a Canadian strain (HMPV 35) isolated in 2001.

The 12 HMPV strains detected in hospitalized children (study population) clearly clustered into two F lineages as previously reported (*1*,*5*,*9*); nine strains belonged to group 1 (which includes the prototype strain from the Netherlands, GenBank accession no. af371337) and three to group 2 (Figure 4). Seven of the nine group 1 strains had identical F gene sequences although they were not temporally related. At the nucleotide level, similarity between groups was 84% to 85%, compared to 98% to 100% within group 1 and 93% to 99% similarity within group 2, respectively.

## Discussion

Our prospective study has shown important clinical and epidemiologic features of HMPV infection. First, our data indicate that HMPV is really a respiratory pathogen with an epidemic behavior. Second, we found that HMPV substantially contributes to ARTI that leads to children’s hospitalization, although in smaller proportion than HRSV and influenza viruses. Although all specimens from this study were tested by PCR, only a subset was studied by viral culture. However, such incomplete virologic testing should not have significantly affected the rate of HMPV infection as evidenced by the absence of additional cases detected by culture. Third, the clinical features associated with HMPV were found to be similar to those of HRSV. Finally, our results suggest that the seasonal pattern of HMPV infection in children may differ from that of HRSV and influenza viruses although additional studies are needed because of our relatively short period of observation.

Recent studies by our group (*8*,*9*) and others (*1*,*4*,*6*,*7*) have suggested that HMPV should be added to the list of human respiratory viral pathogens (*13**–**18*) affecting mainly children, but also other age groups as well. Although the differences in HMPV positivity between our 208 case-patients and 51 controls were not statistically significant (p=0.067), the absence of other respiratory viruses such as HRSV and influenza viruses in 83% of the HMPV-infected children and the severity of the symptoms (bronchiolitis, pneumonitis, or both) suggest that HMPV is a pathogenic respiratory virus. The absence of underlying medical conditions in 75% of the HMPV-infected children further illustrates the pathogenicity of HMPV. The use of PCR was particularly advantageous for HMPV because this virus is fastidious and difficult to grow in most cell lines (*9*); in addition, rapid antigenic detection tests are not currently available.

Our prospective study provides for the first time an estimate of the proportion of ARTI hospitalizations attributable to HMPV in a well-defined pediatric population. From December 15 to April 20, 2002, HMPV was detected in 12 (6%) of 208 children <3 years of age who were hospitalized for respiratory tract infections. This probably underestimates the real impact of this virus because our hospitalization study was stopped before the end of HMPV transmission in the community. However, the percentage of hospitalizations caused by HMPV during the study period was much smaller than that attributable to HRSV or influenza A. Our data are comparable to those of a recent small study from Finland in which HMPV was detected in 8% of children (age range, 4 months to 13.5 years) admitted for acute wheezing (*7*).

Our study found that HMPV disease cannot be distinguished from HRSV and influenza A on clinical findings. However, HMPV disease tended to be somewhat less severe with fewer cases of pneumonia, no admission in the ICU, and a greater proportion of underlying diseases (25%) among infected patients compared with <10% for HRSV or influenza. Nevertheless, HMPV infection was associated with a substantial clinical and economic impact as shown by a median hospital stay of 4.5 days and by the observation that one-third of HMPV-infected case-patients were hospitalized for >7 days.

A small serologic study from the Netherlands showed that all children >5 years of age had HMPV antibodies, which suggests a high level of transmission (*1*). While our study data were limited to children <3 years of age, they suggest that illness caused by HMPV is greatest in children <2 years of age because they represented 10 (83%) of 12 of our hospitalized case-patients and 24 (66%) of 36 of the HMPV isolates recovered in our diagnostic virology laboratory. This finding suggests that, similar to other paramyxoviruses such as HRSV, the illness of HMPV in children occurs through primary infection. In contrast to HRSV, which peaked during the first 2 months of life, HMPV hospitalizations seem to peak in children at a slightly older age, i.e., between the third and fifth month of life. However, given the small number of HMPV cases, this observation needs to be confirmed in a larger study. Should the same trend be observed, this difference may depend on a longer persistence of maternal antibodies or a less efficient transmission mode in the case of HMPV. Both hypotheses would require additional studies.

During the 4-week period from mid-March to mid-April, HMPV infections clustered (11/12 cases) and were associated with 18.9% of all hospitalizations for ARTI in children at our institution. These findings contrast with those for HRSV and influenza A infections, which occurred mostly in January and February. On the basis of passive surveillance data from our regional virology laboratory, the peak time of HMPV transmission in the community occurred between April 6 and 20, 2002, and continued beyond the conclusion of our study in hospitalized children until the end of May. Although incomplete, such data suggest that seasonal outbreaks of HMPV may differ from those of other common respiratory viruses.

As described for HRSV (*12*), several strains of HMPV circulated during a very brief period (1 month) in our study area. The HMPV strains segregated into two F subgroups, in agreement with previous studies (*1*,*5*,*9*), although one strain clearly predominated, accounting for 58.3% of all infections. Because of the small number of HMPV strains belonging to one of the F subgroup, we did not attempt to correlate HMPV genotype with clinical outcome. Such viral heterogeneity may allow multiple reinfections throughout life, especially in elderly persons and immunocompromised patients, as we previously reported (*8*,*9*).

In conclusion, our study supports the concept of the epidemic nature of HMPV infection and its role as a significant pathogen in severe ARTI of children. Year-long active surveillance studies on consecutive years and in different geographic regions are needed to better define the epidemiology of HMPV.
